# Irreversible Electroporation for the Treatment of Pediatric Adenotonsillar Hypertrophy

**DOI:** 10.3390/jcm15135189

**Published:** 2026-07-02

**Authors:** Ulugbek Khasanov, Zukhrob Sobirjonovich Matmuratov, Alexandru Coman, Andrei Mihai Buruiană, Desiderio Passali, Francesco Maria Passali, Ari DeRowe

**Affiliations:** 1 Republican Specialized Scientific and Practical Center of Otorhinolaryngology and Head and Neck Diseases, Tashkent 100084, Uzbekistan; ukhasanov@yahoo.com (U.K.); orlhns.uz@gmail.com (Z.S.M.); 2 “M. S. Curie” Emergency Clinical Hospital for Children, 077120 Bucharest, Romania; alexandrucmn@gmail.com (A.C.); burureal2002@yahoo.com (A.M.B.); 3 ENT Clinic, University of Siena, 53100 Siena, Italy; d.passali@virgilio.it; 4 Department Clinical Sciences and Translational Medicine, University of Rome “Tor Vergata”, 00133 Roma, Italy; passali@med.uniroma2.it; 5 Department of Otolaryngology Head and Neck Surgery, Tel Aviv Sourasky University Medical Center, Gray Faculty of Medical and Health Sciences, Tel-Aviv University, Tel-Aviv 6997801, Israel

**Keywords:** electroporation, tissue ablation, pediatric adenotonsillar hypertrophy, tonsillectomy, adenoidectomy

## Abstract

**Background/Objectives:** Irreversible electroporation (IRE) is a non-thermal tissue ablation technology, offering potential advantages such as reductions in pain, bleeding, and procedural time. With this study, we aimed to evaluate the safety and efficacy of IRE for the treatment of adenotonsillar hypertrophy in pediatric patients. **Methods:** In this prospective, two-center, interventional case series, we enrolled 31 children aged 3–13 years with adenotonsillar hypertrophy and upper airway obstruction. All procedures were performed under general anesthesia. IRE was applied to the tonsils and adenoids using the ENTire™ system. Postoperative pain was recorded daily for seven days using the Parent’s Postoperative Pain Measure. Disease-specific quality of life was assessed at baseline and three months post-procedure using the Obstructive Sleep Apnea questionnaire (OSA-18). Tonsillar size was graded according to the Brodsky Scale. **Results:** The mean procedure time was 10:40 ± 03:40, and there was no intraoperative or postoperative bleeding. Patients were discharged on the day of the procedure. The postoperative pain on day 1 was ranked as 3.5 ± 1.5 and resolved completely in all patients by day 7. From baseline to 3 months, the mean OSA-18 decreased from 66.4 ± 14.9 to 24.6 ± 9.9 (Wilcoxon signed-rank test *p*-value of <0.0001), and the Brodsky grade decreased from 3.26 ± 0.63 to 0.92 ± 0.61 (*p* < 0.001). **Conclusions:** In this preliminary cohort, non-thermal adenotonsillar ablation using irreversible electroporation was safe, well tolerated, and demonstrated short-term improvements in parent-reported symptoms and oropharyngeal tonsillar size in children. These findings support further investigation in larger, multicenter trials with extended follow-ups.

## 1. Introduction

Tonsillectomy is one of the most commonly performed surgical procedures in children, most frequently indicated for upper airway obstruction or recurrent tonsillar infections [1]. In 2019, an estimated 559,900 ambulatory and 7100 inpatient pediatric tonsillectomies were performed in the United States [2]. Despite its frequency and overall safety, postoperative pain and hemorrhage remain clinically significant concerns. In recent years, multiple surgical techniques and technologies have been introduced to mitigate these complications, including intracapsular approaches using coblation or microdebrider systems [3,4,5,6]. Although these less invasive techniques have demonstrated certain advantages, reductions in postoperative pain and bleeding have generally been modest.

Electroporation is a technique that applies pulsed electrical fields to tissue, disrupting cellular ion channels. By delivering high energy levels, the resulting pore formation becomes irreversible, resulting in tissue ablation due to delayed cell death [7]. One of the major advantages of this approach is the ability to precisely target tissue while minimizing damage to surrounding structures. Furthermore, because electroporation is a non-thermal modality, it may decrease complications associated with thermal injury, such as postoperative pain. The electrical pulses are generally administered in microsecond intervals, which contribute to shorter procedure times [7].

Irreversible electroporation (IRE) has been increasingly used for tissue ablation across multiple clinical applications, including the treatment of hepatic tumors, cardiac arrhythmias, and prostate cancer [8,9,10]. A recent study reported the safety and effectiveness of IRE for tonsillar ablation in an adult population [11]. As a non-thermal ablative modality, IRE has the potential to further reduce the risk of damage to adjacent tissues compared with conventional techniques. In addition to the potential reduction in pain, since the tissue is not physically disrupted, there should be negligible bleeding, which represents significant factor to consider in adenotonsillar surgery.

This preliminary study is the first to evaluate IRE technology for non-thermal ablation of the tonsils and adenoids in children. Its primary aim is to assess whether IRE may represent a minimally invasive alternative for adenotonsillar ablation, with the potential to reduce postoperative pain while maintaining procedural safety and clinical effectiveness.

## 2. Materials and Methods

This study was approved by the ethical committee of the Republican Specialized Scientific and Practical Center of Otorhinolaryngology and Head and Neck Diseases and the Ministry of Health, Tashkent, Uzbekistan (Ministry of Health number: 02-10/19966). The approval for the “M. S. Curie” Emergency Clinical Hospital for Children, Bucharest, Romania, was granted under No. 3994/29 January 2025. The bioethical approval for the Romania site was granted by the National Commission for Bioethics of Medicines and Medical Devices, Bucharest, Romania (approval number No. 5DM din 5 March 2025), and the national approval for the Romania site was obtained from the Ministry of Health, National Agency of Medicines and Medical Devices Authority of Romania, Bucharest, Romania (reference No. 96 of 26 May 2025). This study was registered with the National Library of Medicine at the N.I.H. https://clinicaltrials.gov/study/NCT06960239 “Safety and Efficacy of the Irreversible Electroporation (IRE) System for Adenotonsillar Hypertrophy in Children”. Accessed on 16 March 2026.

In this study, thirty-one children aged 3–13 years with adenotonsillar hypertrophy and upper airway obstruction were included, and we excluded children with craniofacial anomalies. Surgical candidacy was determined clinically by the treating physician based on symptoms of sleep-disordered breathing, including habitual snoring, witnessed apneas, increased respiratory effort, sleep disruption, and gasping episodes during sleep.

Informed written consent was obtained from the parents.

At baseline, all participants underwent a physical examination, and tonsil sizes were assessed using the BSGT (Brodsky Scale Grading of Tonsils) [12]. The Brodsky Scale is a pragmatic, validated clinical tool in pediatric cohorts. In addition, the parents of all participants completed the OSA-18, a health-specific quality of life questionnaire [13]. Tonsil grading and questionnaire administration were performed by otolaryngologists who were not involved in the procedure to reduce observer bias.

**Figure 1:**
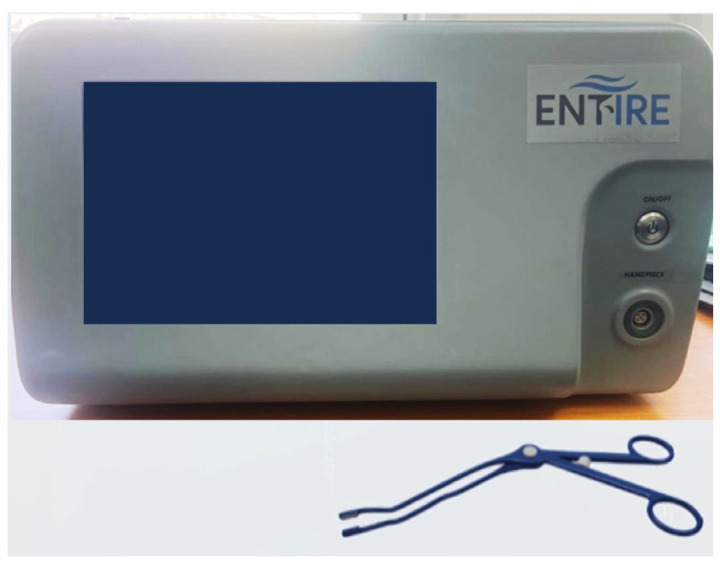
IRE generator + handpiece for tonsils and adenoids (Entire Medical Inc., Richardson, TX, USA).

The procedure was performed under general anesthesia, using the set-up for conventional tonsillectomies with standard mouth gags. The IRE System (Entire Medical Inc. Richardson, TX 7508, USA) consists of a generator (console) and a handpiece designed to deliver high-voltage (1500 volt), microsecond-pulsed electrical power to tissue, generating electric fields that induce the irreversible electroporation effect. The handpiece is designed to act as bi-polar forceps that grab the tissue without penetrating and features two distal electrodes constructed with medical-grade stainless steel, which deliver electrical energy through the tissue surface (Figure 1). Tonsillar ablation was performed under direct visualization and adenoid ablation transorally with soft palate retraction and mirror visualization. For both tonsillar and adenoidal tissue, the bipolar forceps were applied to grasp the tissue at multiple treatment sites, typically 6–8 distinct areas. Activation of the footswitch delivered a train of electrical pulses. Each treatment site was treated 3–6 times, resulting in approximately 40–50 applications per tonsil or adenoid.

Intra-operative bleeding was monitored by estimating blood loss during the procedure. Post-operative bleeding was followed based on return to emergency department or operating room for bleeding control.

Postoperative pain was assessed daily for one week using the Parent’s Postoperative Pain Measure (PPPM) [14]. Analgesia was prescribed on a PRN (as needed) basis, and NSAIDS.

Three months after the procedure, a follow-up visit was conducted, which included a reassessment of the tonsillar size using the BSGT and repeated administration of the OSA-18 questionnaires.

All outcome instruments (PPPM, Brodsky Scale, OSA-18) were selected for their established validity in pediatric populations to minimize subjective variability; however, no assessor was blinded to the treatment status, and all assessments were performed by investigators affiliated with the study sites.

Statistical analysis: All measured variables and derived parameters were tabulated using descriptive statistics. For categorical variables, we provide summary tables that list the sample size, absolute and relative frequency, and 95% CI (Clopper Pearson Confidence Interval) for proportions. For continuous variables, summary tables list the sample size, arithmetic mean, standard deviation, minimum, median, and maximum. The analysis was performed using Excel software.

## 3. Results

Thirty-one otherwise healthy children (mean age: 7.8 ± 3.0 (3.6–13.5) years; 11 female) were included in this study, and all underwent tonsil and adenoid ablation. The mean procedure time was 10:40 ± 03:40 min. There was no intra-operative blood loss and no intraoperative complications. There were no cases of post-operative bleeding. Detailed baseline patient characteristics are provided in Table 1.

**Figure 2:**
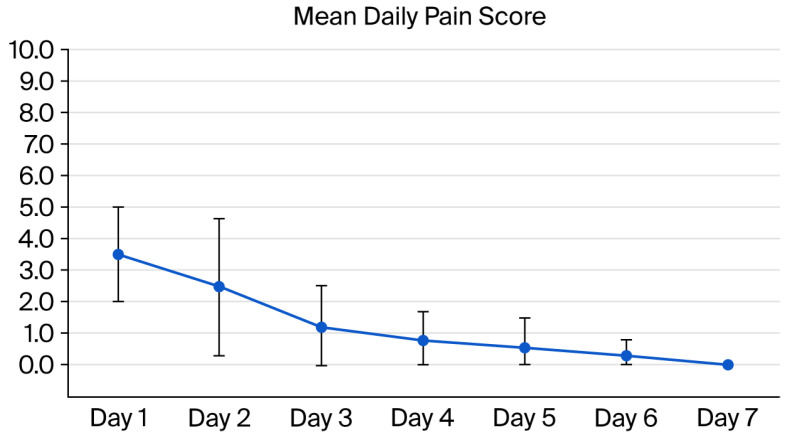
Mean daily scores of the Parent’s Postoperative Pain Measure (PPPM).

The postoperative pain during the first week is shown in Figure 2. Notably, the average pain score on postoperative day 1 was 3.5 ± 1.5. By the end of the first week, no pain was reported. All patients resumed a normal diet on the first postoperative day.

**Figure 3:**
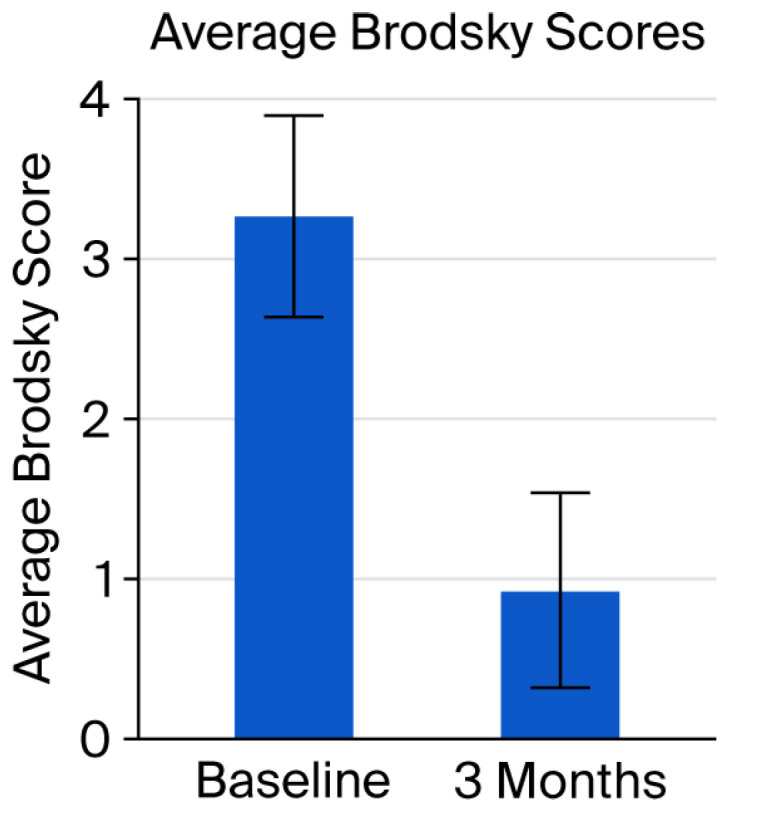
Brodsky Scale Grade of Tonsils (BSGT) for 31 patients (62 tonsils) at baseline and 3 months postoperatively.

As shown in Figure 3, the mean BGST score for the 31 patients was 3.26 ± 0.63, which decreased to 0.92 ± 0.61 at 3 months postoperatively, representing a 71.1% reduction in tonsillar size (Wilcoxon signed-rank test *p* < 0.0001).

**Figure 4:**
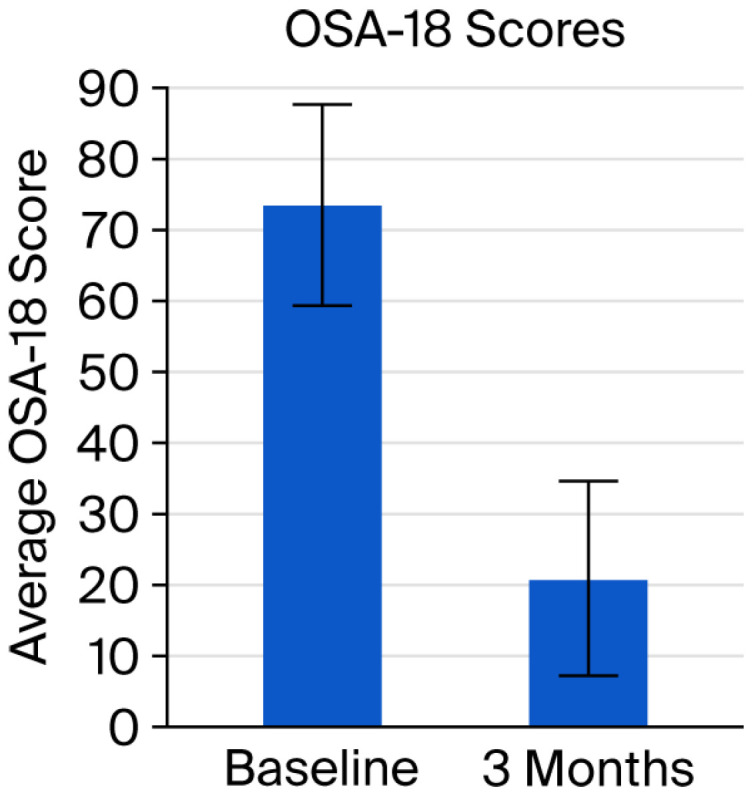
Improvement in OSA-18 scores from baseline to 3 months postoperative.

The change in the OSA-18 scores is presented in Figure 4. The mean OSA-18 score at baseline was 66.4 ± 14.9, which decreased to 24.6 ± 9.9 (Wilcoxon signed-rank test *p* < 0.0001) 3 months postoperatively.

## 4. Discussion

This study represents, to our knowledge, the first report describing the use of irreversible electroporation (IRE) for the non-thermal ablation of tonsils and adenoids in children with upper airway obstruction due to adenotonsillar hypertrophy. Although the sample size was limited, the preliminary results were encouraging. In this series of patients, no intraoperative bleeding occurred, and no postoperative hemorrhages were identified during the routine follow-up; all patients were discharged on the same day. The postoperative pain was minimal with an immediate return to normal diets. After 3 months, a reduction in tonsillar size was observed, which corresponded with clinically meaningful improvements in disease-specific quality of life measures.

IRE is an emerging technology used for the ablation of cardiac arrhythmias and a variety of tumors [15]. Its main advantage over conventional ablative techniques is that it avoids thermal injury, thereby reducing collateral damage to adjacent tissues, including critical neurovascular structures. Additionally, mucosal integrity is largely maintained, which may decrease procedural invasiveness, bleeding, and postoperative discomfort. Histologically, IRE induces delayed apoptosis without disrupting the tissue architecture, resulting in minimal or no bleeding. The present study was conducted as a single-arm feasibility investigation aimed at evaluating procedural safety and obtaining preliminary efficacy data. It was neither designed nor statistically powered to assess comparative effectiveness against established adenotonsillar surgical techniques. Accordingly, the findings should not be interpreted as evidence that IRE is equivalent or superior to conventional approaches. Nonetheless, the findings provide an important foundation for future, larger-scale, multicenter trials. The postoperative course was notable for rapid recoveries. Patients were discharged on the day of the procedure and resumed a normal diet on the first postoperative day. The postoperative pain was minimal, with caregiver-reported postoperative pain on day 1 being 3.5 ± 1.5 and resolved completely in all patients by day 7.

As a first-in-children feasibility study, the efficacy assessment was limited to the oropharyngeal Brodsky grading and validated OSA-18 questionnaire; polysomnography, home sleep apnea testing, and nasopharyngeal imaging were not performed, and the adenoid size was not objectively reassessed at follow-up. Because no objective measure of upper airway patency or sleep-disordered breathing was obtained, and the adenoid component was not reimaged postoperatively, the degree to which the observed symptomatic and oropharyngeal changes reflect a true resolution of airway obstruction—particularly in the nasopharynx—cannot be determined from these data. Although questionnaires are a validated study tool for sleep disordered breathing in children, they are a subjective measure based on parental perception of breathing during sleep and are thus less reliable as an outcome measure compared to polysomnography. Assessments were performed by otolaryngologists who were not involved in the procedure; the use of different evaluators may have introduced inter-observer variability. The absolute reduction in OSA-18 scores (66.4 ± 14.9 to 24.6 ± 9) indicates a large improvement likely to exceed distribution-based thresholds for clinically important changes.

Regarding the tonsillar size reduction, the follow-up period was limited to 3 months. Theoretically, a further reduction in tonsillar size is anticipated, as cellular apoptosis can continue for months in animal models, although the duration of this effect in humans is yet to be determined [16]. Regarding adenoid size reduction, no scoring or measurement was performed pre or post-operatively and thus no conclusion can be made as to the effect on size and symptoms related to adenoid hypertrophy. In a previous study conducted by our group on 24 adult patients treated with similar energy parameters and followed for 3 months, a 58% reduction in tonsillar volume was observed. This was accompanied by significant improvements in disease-specific quality of life scores. The postoperative pain in that cohort was likewise minimal, with a return to normal activity and diets by postoperative day 3 [11].

Long-term outcomes must also be considered. Symptomatic tissue regrowth has been reported, albeit infrequently, following intracapsular tonsillar techniques, with rates of approximately 1–2% over 3–6 years of follow-up [3]. Owing to the short duration of the follow-up in the present study, the potential for late tissue regrowth after IRE could not be assessed and warrants further investigation.

## 5. Conclusions

In summary, IRE ablation of the tonsils and adenoids in children represents a promising and innovative approach that may provide a less invasive alternative to existing surgical techniques. Large-scale multicenter studies are currently underway to further assess the safety, efficacy, durability of tissue reduction, and long-term clinical outcomes associated with the treatment of pediatric tonsillar and adenoidal disease using IRE.

## Figures and Tables

**Table 1:** Patient characteristics.

Parameter (Unit)	Mean ± SD (Range) (Where Applicable)
Age (years)	7.8 ± 3.0 (3.6–13.5)
Height (cm)	125.1 ± 20.2 (90.0–160.0)
Weight (kg)	31.8 ± 16.1 (13.0–77.0)
Sex (*n* (%))	(*n* (%))
Male	20 (65)
Female	11 (35)
Baseline Tonsil Size (BGST) (*n* (%))	*n* (%)
Left Tonsil	
Grade 0	0 (0)
Grade 1	0 (0)
Grade 2	3 (10)
Grade 3	17 (55)
Grade 4	11 (35)
Right Tonsil	
Grade 0	0 (0)
Grade 1	0 (0)
Grade 2	3 (10)
Grade 3	17 (55)
Grade 4	11 (35)

## Data Availability

https://clinicaltrials.gov/study/NCT06960239 “Safety and Efficacy of the Irreversible Electroporation (IRE) System for Adenotonsillar Hypertrophy in Children”. Accessed on 16 March 2026.
